# Effect of nano-SiO_2_ and nano-CaCO_3_ on the static and dynamic properties of concrete

**DOI:** 10.1038/s41598-021-04632-7

**Published:** 2022-01-18

**Authors:** Zhi-hang Wang, Er-lei Bai, Jin-yu Xu, Yu-hang Du, Jing-sai Zhu

**Affiliations:** 1 Department of Airfield and Building Engineering, Air Force Engineering University, Xi’an, 710038 China; 2 College of Mechanics and Civil Architecture, Northwest Polytechnic University, Xi’an, 710072 China

**Keywords:** Engineering, Materials science, Nanoscience and technology

## Abstract

Three kinds of nano-concrete, i.e., 2.0% nano-SiO_2_ doped, 2.0% nano-CaCO_3_ doped and 1.0% nano-SiO_2_-1.0% nano-CaCO_3_ co-doped concretes (NS, NC, NSC) were prepared for a study on static property and dynamic property under different strain rates (50–130 s^−1^) using HYY series hydraulic servo test system and Φ100 mm split Hopkinson pressure bar test system, and a comparison with plain concrete (PC) as well. The results have shown that under static load, as compared with PC, NC has both strength and elastic modulus increased obviously, while NS has strength decreased and elastic modulus increased, and under dynamic load, there is an obvious strain rate effect for the dynamic compressive strength, impact toughness, energy dissipation and impact failure mode of concrete. Under the same strain rate, the dynamic compressive strength, peak strain, impact toughness and energy dissipation of NC are significantly increased, while its dynamic elastic modulus is decreased. Compared with PC, NS has dynamic compressive strength, peak strain, impact toughness and energy dissipation decreased, and dynamic elastic modulus increased, NC has static and dynamic mechanical properties improved, NS has static and dynamic mechanical properties weakened, and NSC is between PC and NC in static and dynamic mechanical properties, but generally improved. Doped with nano-CaCO_3_, NC has compactness improved, weak areas reduced, and pore size distribution optimized, while doped with nano-SiO_2_, NS has obvious internal weak areas, with pore structure degraded.

## Introduction

In the construction industry, concrete has become the most widely adopted building material with the maximum consumption in human history because it is convenient, economical, and practical, and it is irreplaceable in engineering^1–3^. With the continuous expansion of concrete structure in scale and scope of use, its service environment and load are more and more complicated^4,5^. Therefore, considering the safety and reliability of concrete structure, it is necessary to adapt it to the increasingly harsh engineering conditions through relevant modifications^6–8^. Ultrafine powders, represented by nanomaterials, are a new material used for the modification of concrete^9,10^. The mechanical and deformation properties of concrete may be improved effectively after adding the nanomaterials into its matrix^11,12^.

At present, the research on nano-concrete has been very fruitful, especially on nano-SiO_2_ modified concrete and nano-CaCO_3_ modified concrete^13–15^. Abreu G B D et al. found that nano-SiO_2_ added in high performance water-reducing agent of polycarboxylates was conductive to uniform dispersion of the agent in concrete. An appropriate amount of nano-SiO_2_ can improve the microstructure of concrete, thus increasing the compressive strength and elastic modulus of concrete^16^. Shaikh et al.^17^ studied the effect of nano-SiO_2_ on the mechanical properties of recycled coarse aggregate concrete, and found that nano-SiO_2_ can improve the compressive strength of recycled coarse aggregate concrete, in other words, if concrete contains 25% recycled coarse aggregate and 2.0% nano-SiO_2_, its compressive strength could reach 92% of the normal compressive strength of concrete. The study of Sivasankaran^18^ et al. concerning the influence of nano-SiO_2_ on the performance of concrete mixed with coal ash showed that after added with 1.0% nano-SiO_2_, the concrete containing 25% coal ash had a compressive strength increased by 23% and a tensile strength increased by 28%. Li et al.^19^ found that 2.0% nano-CaCO_3_ could enhance the compactness and mechanical properties of concrete, but excessive nano-CaCO_3_ led to local defects poor mechanical properties of concrete. Camiletti J et al. studied the influence of nano-CaCO_3_ on the early mechanical properties of ultra-high-performance concrete, and found that nano-CaCO_3_ promoted the early solidification and hardening of concrete. Specifically, 5–10% of nano-CaCO_3_could improve the mechanical properties of concrete effectively^20^. Most of above studies focused on static mechanical properties of nano-SiO_2_ modified concrete and nano-CaCO_3_ modified concrete^21,22^, that is, there are few studies concerning relevant dynamic mechanical properties. However, besides static load, many concrete structures will inevitably be threatened by dynamic loads^23,24^, e.g., impact, explosion and etc. during their service period^25–27^. Therefore, it is necessary to study the mechanical properties of nano-concrete under dynamic load^28^. In addition, the nano-SiO_2_ and nano-CaCO_3_ composite modified concrete is to be further explored, for there are few relevant reports.

In this study, nano-SiO_2_ and nano-CaCO_3_ are used as materials for modification and high-performance water-reducing agent of polycarboxylates as dispersant of nanomaterial and water reducing agent of concrete for preparing 3 kinds of nano-concrete (NS, NC, NSC), which were nano-SiO_2_ doped, nano-CaCO_3_ doped, and nano-SiO_2_/nano-CaCO_3_ co-doped respectively. HYY series hydraulic servo test system and Φ100 mm split Hopkinson pressure bar (SHPB) test system were utilized to study the static and dynamic properties of the three kinds of nano-concrete under different strain rates, and have a comparison with plain concrete (PC) based on indexes of static mechanical performance like stress–strain curve, compressive strength, flexural strength, splitting tensile strength, elastic modulus, and indexes of dynamic mechanical performance like compressive strength, peak strain, dynamic modulus of elasticity, impact toughness, impact energy dissipation, and impact failure mode. The effects of nano-SiO_2_ and nano-CaCO_3_ on the static and dynamic mechanical properties of the concretes were analyzed, and relevant microscopic mechanism was explored by scanning electron microscopy and mercury injection test.

## Experiments

### Materials

**Figure 1:**
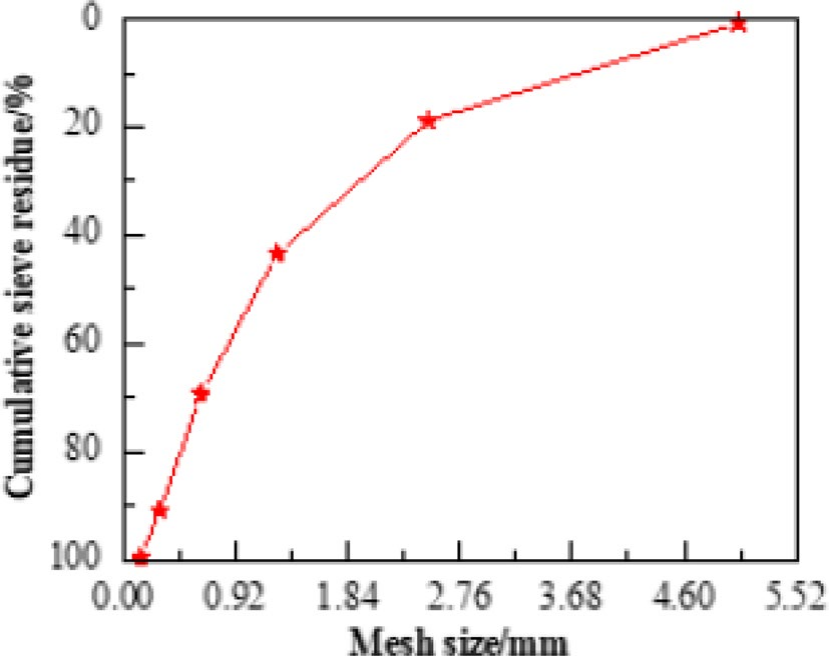
The sand grading curve.

**Figure 2:**
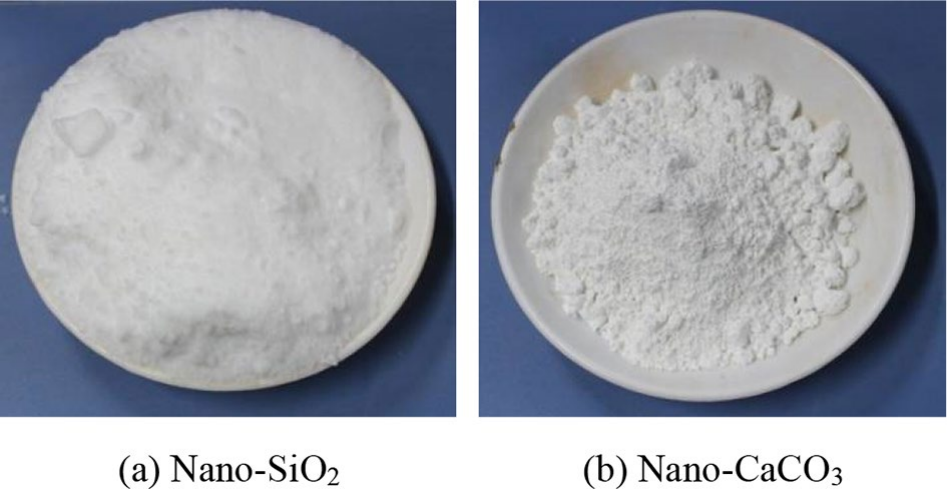
Two kinds of nanomaterials.

**Figure 3:**
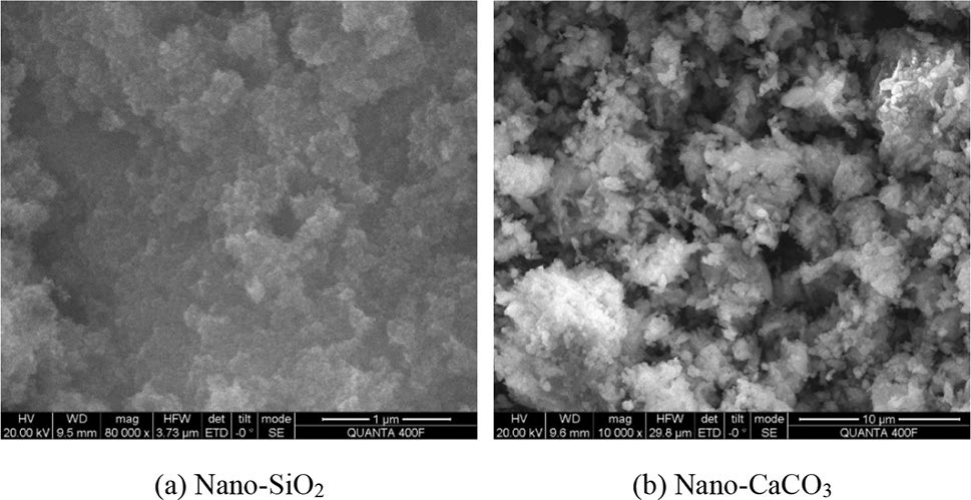
The SEM of two kinds of nanomaterials.

Cement: Qinling PC32.5R cement, Yao County, Shaanxi. The main properties is shown in Table 1, and the chemical composition is shown in Table 2. Coal ash: Low calcium Grade I coal ash produced by Hancheng Power Plant. Fine aggregate: volume weight: 2730 kg/m^3^, fineness modulus: 2.68, bulk density: 1450 kg/m^3^, and silt content: 1.0% of Ba river medium sand. The sand grading curve is shown in Fig. 1. Coarse aggregate: volume weight 2750 kg/m^3^ of limestone gravel (5–10 mm, 15%; 10–20 mm, 85%). Water-reducing agent: mother liquor of high-performance water-reducing agent of polycarboxylates of 40% solid content produced by Shaanxi Zhongyi Chemical Company, used as water-reducing agent for concrete and dispersant for nanoparticle. The main performance indexes are shown in Table 3. Nanoparticles: nano-SiO_2_ and nano-CaCO_3_ produced by Hangzhou Wanjing Company, as shown in Fig. 2, the SEM of two kinds of nanomaterials are shown in Fig. 3. For the main performance indexes, see Table 4.

**Table 1:** Main properties of cement.

Cement type	Specific surface area/(m^2^/kg)	Standard consistency/%	Setting time/min		Stability	3 d strength/Mpa	
			Initial setting	Final setting		Flexural strength	Compressive strength
PC32.5R	486	26.2	129	209	Qualified	4.3	19

**Table 2:** Chemical composition of cement.

Chemical composition	SiO_2_	CaO	Al_2_O_3_	Fe_2_O_3_	f-CaO	MgO	SO_3_
%	23.16	64.85	5.64	4.63	0.28	1.68	–

**Table 3:** Main performance indexes of mother liquor for high performance water-reducing agent of polycarboxylates.

Water-reducing rate/%	Secrete water rate/%	Gas content /%	Difference in setting time/min	Compressive strength rate/%		Shrinkage ratio/%	Appearance
				7d	28d		
≥ 25	≤ 60	≤ 6.0	− 210	≥ 150	≥ 140	≤ 110	Brown clear liquid

**Table 4:** Main performance indexes of the two kinds of nanoparticles.

Nanomaterial	Model	Appearance	Content/%	Specific surface area/(m^2^/g)	Diameter/nm	Crystal form	Water content/%
Nano-SiO_2_	VK-SP15	White flocculent powder	≥ 99.8	250 ± 30	10–20	α	≤ 0.1
Nano- CaCO_3_	VK-Ca01	White powder	≥ 95.0	180 ± 30	40	Cube	≤ 0.5

### Specimens preparation

Concrete specimens were prepared according to the mix ratios as shown in Table 5, where PC represents plain concrete, NS, 2.0wt% nano-SiO_2_ doped concrete, NC, 2.0wt% nano-CaCO_3_ doped concrete, and NSC, 1.0wt% nano-SiO_2_-1.0wt% nano-CaCO_3_ co-doped concrete.

**Table 5:** Mix ratio of concrete (unit: kg/m^3^).

Specimen number	Cement	Coal ash	Gravel	Sand	Water	Water reducing agent	Nano-SiO_2_	Nano-CaCO_3_
PC	396	132	1360	643	215	7.92	–	–
NS	396	132	1360	643	215	7.92	10.56	–
NC	396	132	1360	643	215	7.92	–	10.56
NSC	396	132	1360	643	215	7.92	5.28	5.28

The concrete preparation process is as follows: (1) Mix the water-reducing agent with 3/4 water, stir until dissolution (30 s), and then, add nanoparticles into the solution. After high-speed mixing (30 s, 200 r/min), perform ultrasonic dispersion (15 min) to make a mixture of nanoparticles. (2) Mix the coal ash and 1/2 cement until a uniform ash mixture is formed (30 s). (3) Add the mixture of nanoparticles to the ash mixture, and stir until a mortar is formed (30 s). (4) Add the sand and gravel successively into the mortar, and stir (60 s). (5) Add the remaining 1/4 of water and 1/2 of cement, and stir until a uniform mixture is formed (120 s), that is, fresh concrete.

**Figure 4:**
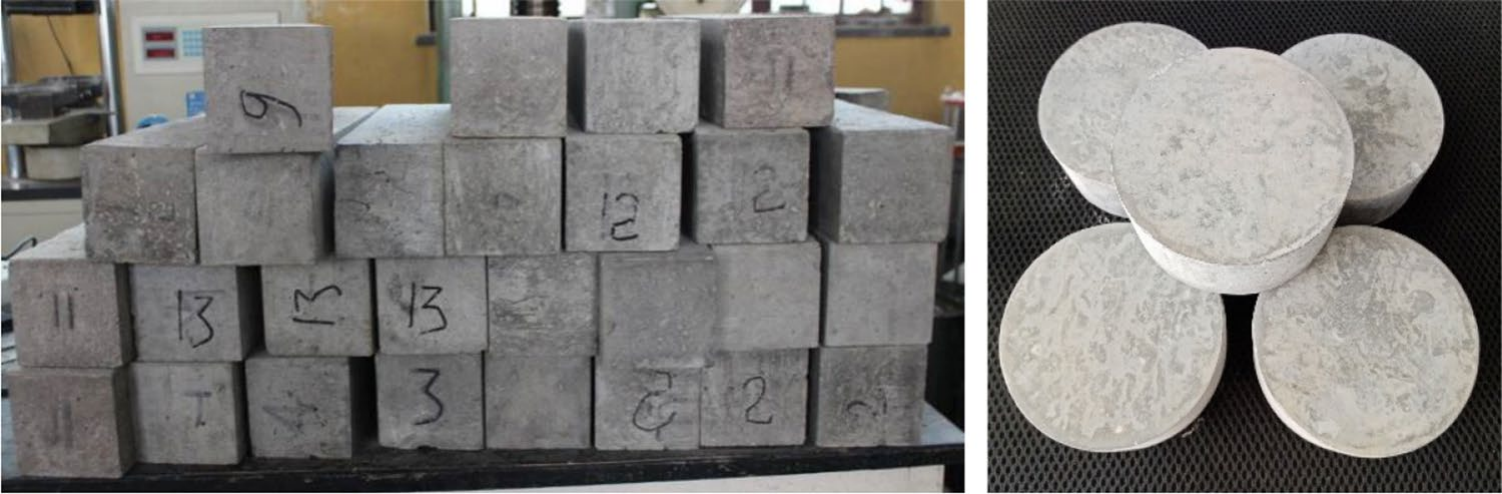
Concrete specimen.

The fresh concrete is cast in a mold. After 24 h of curing in a standard curing box (20 °C ± 2 °C, RH ≥ 95%), the mold is removed. Then, the mold is further cured for 28 days. In the static mechanical test, the dimensions of specimen for concrete compression test and splitting tensile test of concrete are 100 mm × 100 mm × 100 mm, and for flexural test of concrete is 100 mm × 100 mm × 400 mm. The dimensions of specimen for dynamic mechanical test are Φ100 mm × 50 mm. The specimens are finely polished by double—end grindstone machine, and the final test specimen is a cylinder of diameter 99 mm ± 1 mm and length 49 mm ± 1 mm, with surface nonparallelism controlled within 0.02 mm. Some concrete specimens are as shown in Fig. 4.

### Test methods

**Figure 5:**
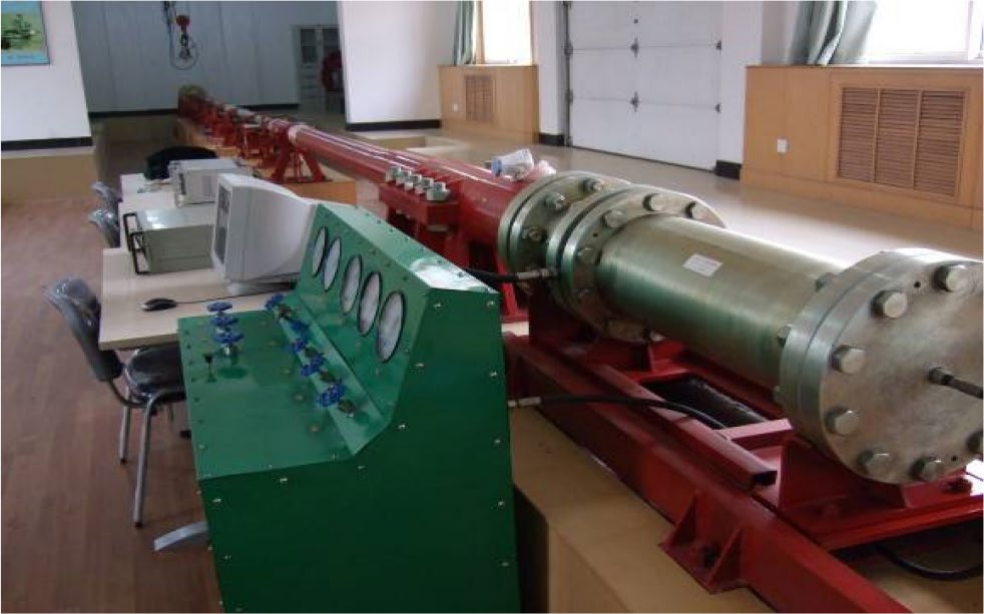
Φ100 mm SHPB test system.

In the static mechanical test, the HYY electro-hydraulic servo material test system was used for the compressive test, bending test, and splitting tensile test of concrete. The dynamic mechanical test was performed with Φ100 mm split Hopkinson pressure bar (SHPB) test system as shown in Fig. 5.

**Figure 6:**
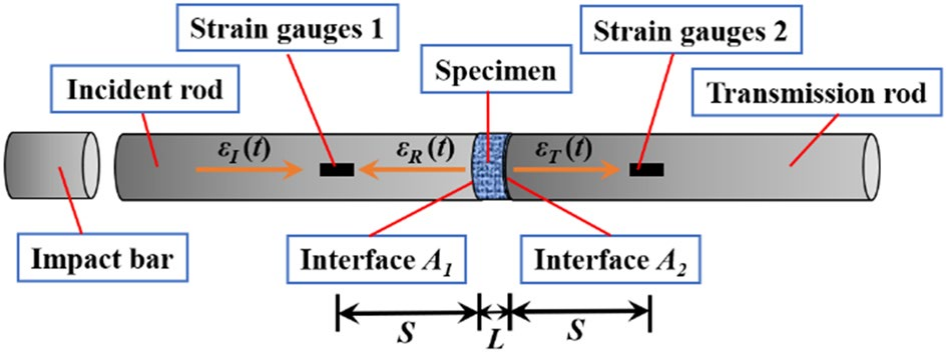
Schematic diagram of stress wave propagation in dynamic mechanical test.

The propagation principle of stress wave for the SHPB test system was as shown in Fig. 6. The input compressed air provided an initial velocity for the impact bar, and the impact of the incident bar produces *ε*_*I*_(*t*), the incident stress wave, part of which was reflected to form a reflected stress wave *ε*_*R*_(*t*) as passing through interface A1, while another part formed transmitted stress wave *ε*_*T*_(*t*) as passing from the specimen through interface A2 to the transmission bar. The incident stress wave and the reflected stress wave were measured by strain gage 1, and the transmitted stress wave is measured by strain gage 2.

The "three-wave method" was used to process the test data and calculate the strain, strain rate and stress of specimen, i.e., *ε*_*s*_(*t*), $\overline{\dot{\varepsilon }}_{s} \left( t \right)$ and *σ*_*s*_*(t)*^29^.

1 $$ \begin{aligned} \varepsilon_{s} \left( t \right) & = \frac{{C_{e} }}{L}\mathop \smallint \limits_{0}^{\tau } \left[ {\varepsilon_{I} \left( t \right) - \varepsilon_{R} \left( t \right) - \varepsilon_{T} \left( t \right)} \right]dt \\ \overline{\dot{\varepsilon }}_{s} \left( t \right) & = \frac{{C_{e} }}{L}\left[ {\varepsilon_{I} \left( t \right) - \varepsilon_{R} \left( t \right) - \varepsilon_{T} \left( t \right)} \right] \\ \sigma_{s} \left( t \right) & = \frac{{E_{e} A_{e} }}{{2A_{s} }}\left[ {\varepsilon_{I} \left( t \right) + \varepsilon_{R} \left( t \right) + \varepsilon_{T} \left( t \right)} \right] \\ \end{aligned} $$

where C_*e*_ represents the propagation velocity of stress wave in the bar, *L*, the thickness of specimen, *τ*, the propagation time of stress wave in the bar, *E*_*e*_, *A*_*e*_, elastic modulus and cross-sectional area of the bar respectively, *A*_*s*_, the area of specimen end face.

**Figure 7:**
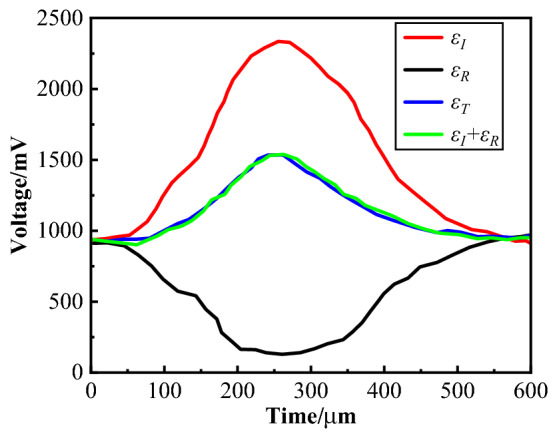
Typical waveforms.

Typical waveforms measured by the test were shown in Fig. 7. Obviously, *ε*_*T*_*(t)* had good coincidence with *ε*_*I*_*(t)* + *ε*_*R*_*(t)* in waveform, indicating that the specimen was in a state of dynamic stress equilibrium during impact loading.

For the SEM test, COXIEM-30 scanning electron microscope was used to observe the microstructure of concrete. The samples were fragments after the mechanical performance testing, and the samples were processed by a particle sputtering machine before testing. In the MIP test, the pore structure of concrete was analyzed by the PoreMaster Mercury Injection Apparatus. After normal curing, the samples underwent 1d of low-temperature drying at a temperature of 50 °C, and for the samples, the mass was accurately weighed before the mercury injection test.

## Results and analysis

### Static mechanical properties

#### Stress–strain curve

**Figure 8:**
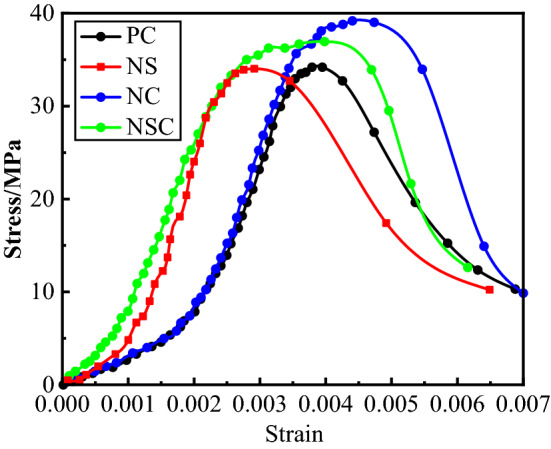
Stress–strain curve of concrete under static compression load.

The stress–strain curve may comprehensively reflect the properties and indexes of material, and the changes of geometric features and feature points may directly reflect the strength and deformation characteristics of a specimen at different stress stages. The stress–strain curve of concrete under static compression load was shown in Fig. 8. Obviously, under the quasi-static compression load, the four groups of concrete first underwent a process of compaction in the early phase of stress, in which the strain increased with stress.

The peak stress of NC and NSC was significantly higher than that of PC, and the peak stress of NC was the largest, while the peak stress values of NS and PC were not significantly different, indicating that the addition of nano-SiO_2_ had no obvious effect on the peak stress of concrete, while the addition of nano-CaCO_3_ had. As compared with PC, NS, NC and NSC were near the peak stress, and their curves were approximate a plateau, especially for NSC, indicating that NS, NC and NSC still maintain a certain strength in a large range with the increase of strain near the peak stress until the specimens lost their bearing capacity after failure, i.e., the addition of nano-SiO_2_ or nano-CaCO_3_ improved the deformation performance of concrete.

#### Compressive strength, flexural strength, and splitting tensile strength

**Figure 9:**
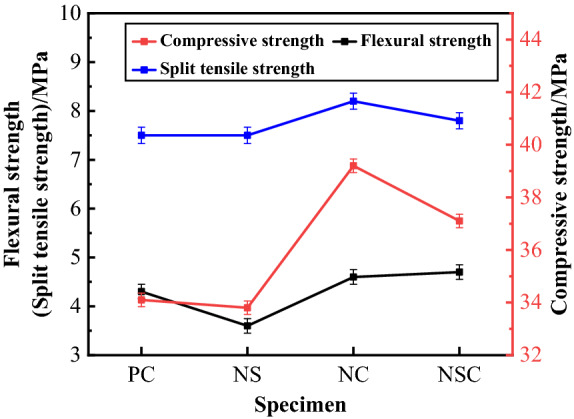
Compressive strength, flexural strength and splitting tensile strength of concrete.

The compressive strength, flexural strength, and splitting tensile strength of concrete are as shown in Fig. 9. As can be seen from the figure, compared with PC, NC and NSC had compressive strength increased significantly, which was up to 39.2 MPa and 37.1 MPa, or increased by 14.96% and 8.80% respectively. The compressive strength of NS decreased by 0.88%. The flexural strength of PC was 4.3 MPa, of NC and NSC was increased to 4.6 MPa and 4.7 MPa respectively, but of NS was significantly reduced by 16.28%. NS and PC had the same splitting tensile strength, 7.5 MPa. NC had the largest splitting tensile strength, which increased by 9.33% as compared with PC. NSC also had an increased splitting tensile strength, which was increased by 4.00%. The nano-CaCO_3_ significantly improved the strength of concrete, but nano-SiO_2_ was not so good, or even compromised the strength of concrete. The nano-CaCO_3_/nano-SiO_2_ co-doping also improved the strength, but to a limited extent. The strengthening effect of nano-CaCO_3_ was partly offset by nano-SiO_2_.

#### Elastic modulus

**Figure 10:**
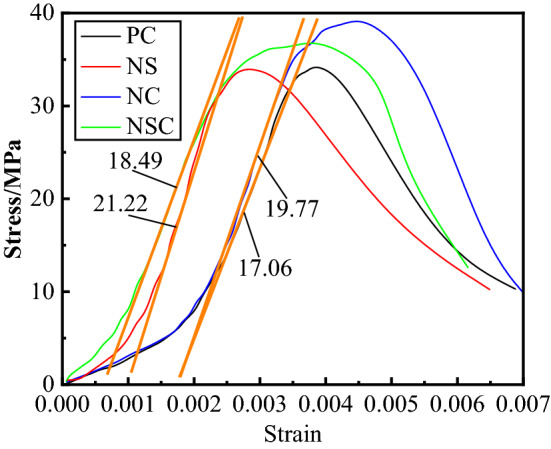
Elastic modulus of concrete.

The ability of concrete to resist elastic deformation can be expressed as elastic modulus. The commonly used expression modes for elastic modulus include initial elastic modulus, tangent modulus, secant modulus, mixed modulus, and etc., which have different meanings. Based on the stress–strain curve under the quasi-static compression load, the concrete samples mixed with different nanoparticles were different greatly in the ability to resist elastic deformation. In this study, the mixed elastic modulus was used for the expression as follows:

2 $$ E_{c,s} = \frac{{0.6f_{p,s} - 0.4f_{p,s} }}{{\varepsilon_{0.6} - \varepsilon_{0.4} }} $$

where $f_{p,s}$ represents the peak stress; $\varepsilon_{0.6}$, $\varepsilon_{0.4}$, strains corresponding to $\varepsilon_{0.6} ,\varepsilon_{0.4}$ in the stress–strain curve respectively. The elastic modulus of concrete was as shown in Fig. 10. Obviously, the elastic modulus of PC was the smallest, which was only 17.06 GPa, after mixed with nano-SiO_2_ or nano-CaCO_3_, the elastic modulus of concrete increased to greater than 18 GPa. The elastic modulus of NS was the largest, which was 21.22 GPa. As compared with NS and NC, the elastic modulus of NSC decreased, indicating that nano-SiO_2_ and nano-CaCO_3_ interacted in the concrete, thus inhibiting the strengthening effect mutually.

### Dynamic mechanical properties

#### Dynamic compressive strength

**Figure 11:**
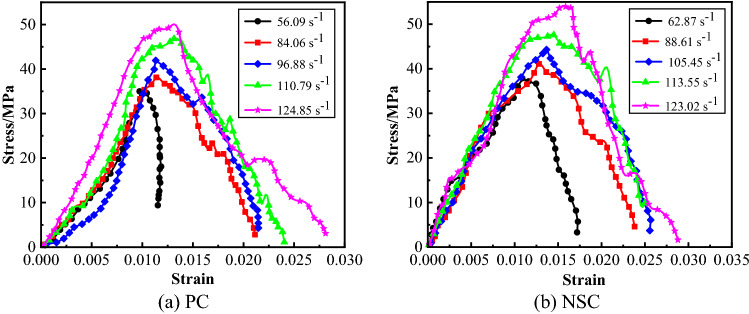
Stress–strain curves of concretes under dynamic load.

The stress–strain curves of some concretes under dynamic load were as shown in Fig. 11. many critical parameters of dynamic mechanical performance can be obtained based on the stress–strain curves of concretes under dynamic load. For details, see Table 6.

**Table 6:** SHPB test results of concrete.

Sample	Serial number	Mean strain rate	Dynamic compressive strength	Strength increase factor	Peak strain	Dynamic elastic modulus	Impact toughness	Energy dissipation
		$\overline{\dot{\varepsilon }}$/(s^-1^)	$f_{d}$/(MPa)	*I*	$\varepsilon_{d}$	$E_{d}$/GPa	KJ/m^−3^	KJ
PC	1	56.09	37.34	1.065	0.0104	5.73	0.206	97.63
	2	84.06	38.77	1.138	0.0113	4.89	0.230	144.65
	3	98.39	42.94	1.260	0.0114	6.52	0.258	155.32
	4	110.79	46.87	1.376	0.0129	5.82	0.296	183.49
	5	124.84	49.81	1.462	0.0131	4.99	0.269	264.47
NS	1	67.52	35.93	1.06	0.0134	4.29	0.189	101.72
	2	95.93	39.51	1.17	0.0129	4.46	0.213	118.11
	3	108.56	42.22	1.25	0.0121	5.04	0.238	183.14
	4	115.4	44.63	1.32	0.0107	7.86	0.288	253.47
	5	125.32	47.54	1.41	0.0101	8.42	0.275	269.42
NC	1	58.86	39.77	1.02	0.011	6.65	0.220	111.54
	2	66.05	43.1	1.10	0.0122	4.16	0.260	181.45
	3	89.34	46.53	1.19	0.0148	3.59	0.379	249.37
	4	112.37	49.64	1.27	0.0157	3.39	0.480	299.22
	5	121.18	53.79	1.38	0.0164	3.47	0.546	342.57
NSC	1	62.87	38.18	1.03	0.0111	3.46	0.234	147.55
	2	88.61	41.37	1.11	0.0129	5.31	0.296	210.11
	3	105.45	44.41	1.21	0.0131	3.52	0.319	232.56
	4	113.55	48.93	1.32	0.0139	3.89	0.397	256.71
	5	123.02	54.02	1.45	0.0151	5.05	0.466	298.44

**Figure 12:**
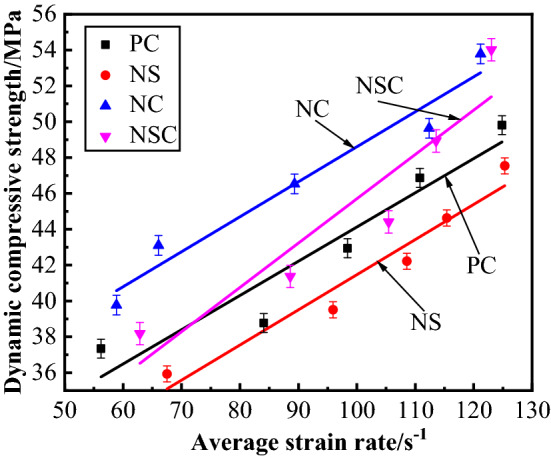
Relationship between dynamic compressive strength and average strain rate of concrete.

The dynamic compressive strength of concrete is the peak stress on the stress–strain curve of concrete under dynamic load, and the limit strength of concrete when it was damaged under dynamic load as well. It is an important parameter representing the bearing capacity of concrete under dynamic load^27^. The relationship between concrete dynamic compressive strength and average strain rate was as shown in Fig. 12. Formula (3) of linear relation was obtained through fitting the dynamic compressive strength with the average strain rate, Based on Fig. 10 and Formula (3), the average strain rate was 50–130 s^−1^. For the four kinds of concrete, the dynamic compressive strength increased with the average strain rate. Under the same level of average strain rate, the dynamic compressive strength of NC was the largest, indicating that the addition of nano-CaCO_3_ increased the dynamic compressive strength of concrete significantly. As a whole, the fitting curve for dynamic compressive strength of NS was below that of PC, indicating that the addition of nano-SiO_2_ decreased the dynamic compressive strength of concrete. When the average strain rate was low, the dynamic compressive strength of NSC was smaller than that of PC, and when the average strain rate was high, the dynamic compressive strength of NSC was larger than that of PC, and the nano-CaCO_3_–nano-SiO_2_ co-doping improved the dynamic compressive strength of concrete at high strain rate. At the same average strain rate level, the dynamic compressive strength of NC was larger than that of NSC, and in improving the dynamic compressive strength of concrete, the effect of separate nano-CaCO_3_ doping was better than that of nano-CaCO_3_–nano-SiO_2_ co-doping.

3 $$ \left\{ {\begin{array}{*{20}c} {\begin{array}{*{20}c} {f_{d - PC} = 25.027 + 0.191\overline{\dot{\varepsilon }}} \\ {f_{d - NS} = 21.856 + 0.196\overline{\dot{\varepsilon }}} \\ {f_{d - NC} = 29.082 + 0.195\overline{\dot{\varepsilon }}} \\ \end{array} } \\ {f_{d - NSC} = 20.973 + 0.247\overline{\dot{\varepsilon }}} \\ \end{array} } \right. $$

**Figure 13:**
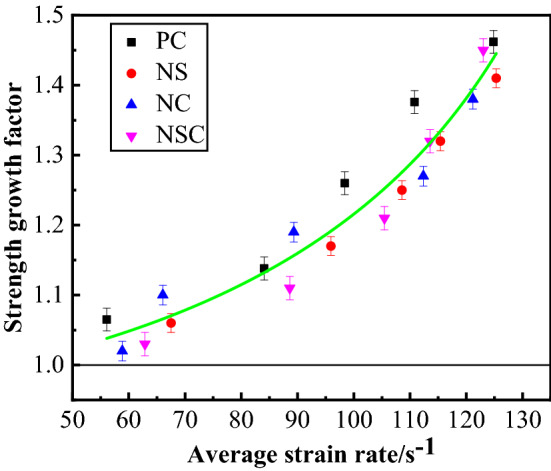
Relationship between strength and average strain rate of concrete.

*I* represents the strength increase factor of concrete under dynamic load, which reflects the enhancement effect of dynamic load on the strength of concrete. Relevant formula is as follows:

4 $$ I = {\raise0.7ex\hbox{${f_{d} }$} \!\mathord{\left/ {\vphantom {{f_{d} } {f_{s} }}}\right.\kern-\nulldelimiterspace} \!\lower0.7ex\hbox{${f_{s} }$}} $$

where $f_{d}$ is the dynamic compressive strength, and $f_{s}$, the compressive strength under static load. The relationship between concrete strength increase factor and average strain rate was as shown in Fig. 13. Obviously, the strength increase factors of the four kinds of concrete were all sensitive to strain rate. With the increase of average strain rate, the greater the increase of dynamic compressive strength, the greater the strength increase factor. The strength increase factors of concretes were all greater than 1, and the dynamic compressive strength was greater than the compressive strength under static load for all. Under the same average strain rate, there was no significant difference between the four kinds of concrete in strength increase factor.

#### Peak strain

**Figure 14:**
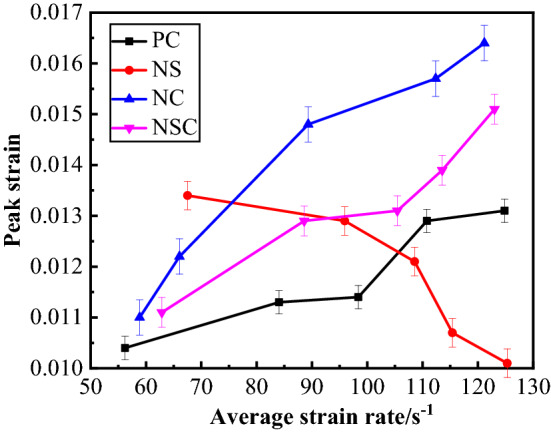
Relationship between peak strain and average strain rate of concrete.

The peak strain refers to the strain corresponding to the peak stress of concrete under dynamic load. At this point, the concrete is at the critical value for unstable crack propagation, that is, any further loading will damage the concrete and make it unable to bear effective load any more^30^. Therefore, peak strain is an important parameter representing the compressive deformability of concrete under dynamic load. The relationship between peak strain and average strain rate of concrete is as shown in Fig. 14. Obviously, when the average strain rate is 50–130 s^−1^, there were significant differences between the four kinds of concrete in the trend of peak strain. With the increase of the average strain rate, for PC, NC and NSC, the peak strain increased, while for NS, the peak strain decreased. Under the same average strain rate, as compared with PC, the peak strain of NC and NSC increased significantly, especially that of NC increased more obviously. The nano-CaCO_3_ can improve the deformability of concrete under dynamic load, while nano-CaCO_3_/nano-SiO_2_ co-doping may decrease the deformability of concrete under dynamic load. When the average strain rate was less than 70 s^−1^, the peak strain of NS was at maximum, and when the average strain rate was greater than 110 s^−1^, the mean strain rate of NS was at minimum. At a low strain rate level, the nano-SiO_2_ can improve the deformability of concrete, while at a high strain rate level, the nano-SiO_2_ will weaken the deformability of concrete.

#### Dynamic elastic modulus

**Figure 15:**
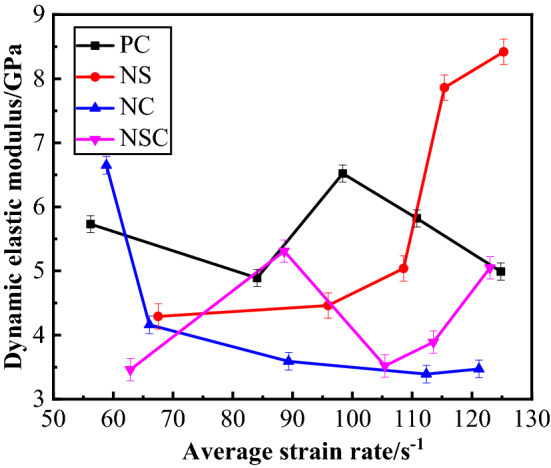
Relationship between dynamic elastic modulus and average strain rate of concrete.

The calculation method of dynamic elastic modulus of concrete is consistent with that of elastic modulus under static load, and the formula is as follows:

5 $$ E_{d} = \frac{{0.6f_{d} - 0.4f_{d} }}{{\varepsilon_{0.6} - \varepsilon_{0.4} }} $$

where $\varepsilon_{0.4} ,\varepsilon_{0.6}$ represent the corresponding strains corresponding to $0.4f_{d} ,0.6f_{d}$ in the stress–strain curve of concrete under dynamic load respectively. The relationship between dynamic elastic modulus and average strain rate concrete is as shown in Fig. 15. Obviously, the dynamic elastic modulus values of concrete at different average strain rate levels were very different. The dynamic elastic modulus of PC was relatively stable, fluctuated around 5.5 GPa, with maximum value 6.52 GPa and minimum value 4.89 GPa. The dynamic elastic modulus of NS increased with the average strain rate. At a high strain rate level, the dynamic elastic modulus of NS was the biggest among the four kinds of concrete, which was up to 8.42 GPa. With the increase of the average strain rate, the dynamic elastic modulus of NC decreased, but at last became stable. At a low strain rate level, the dynamic elastic modulus of NC was the biggest among the four kinds of concrete, which was up to 6.65 GPa. Generally, the dynamic elastic modulus of NSC was smaller than that of PC, and fluctuated around 4.5GPa. The nano-CaCO_3_ can improve the dynamic elastic modulus of concrete at a low strain rate level, while nano-SiO_2_ can improve the dynamic elastic modulus of concrete at a high strain rate level. And the nano-CaCO_3_/nano-SiO_2_ co-doping may decrease the dynamic elastic modulus of concrete.

#### Impact toughness

**Figure 16:**
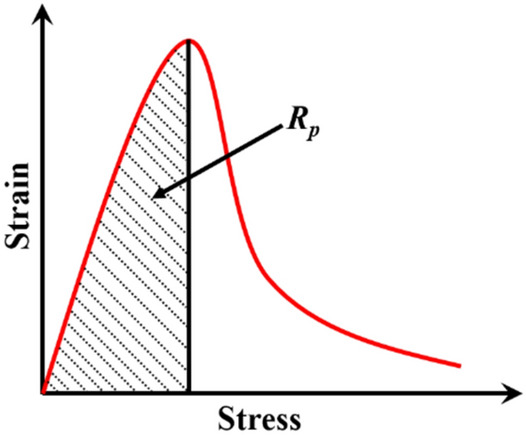
Schematic diagram for the impact toughness of concrete.

The stress–strain curve represents the whole process of concrete specimen from loading to failure, and it may reflect the toughness of the specimen to a great extent. Figure 16 is the stress–strain curve for the general process of failure under load of concrete, in which the section before the peak stress indicates that the concrete specimen enters a unstable fracture propagation stage at the peak stress after the compaction stage, the elastic compression stage, and the fracture propagation stage^31^. Thus, the curve before the peak stress reflects the complete bearing process of concrete specimen before the destabilization. The physical significance of impact toughness $R_{p}$ is the area enclosed by the stress–strain curve and strain axis before the peak stress is reached.

**Figure 17:**
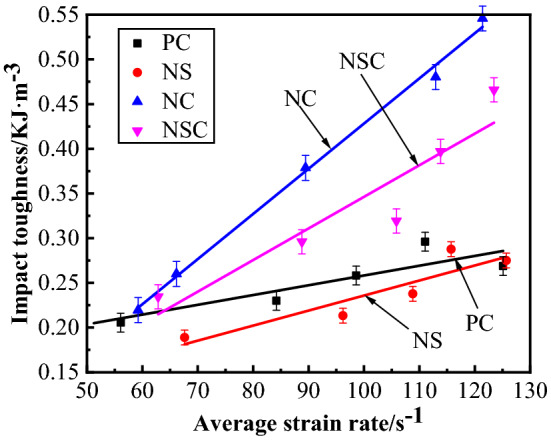
Relationship between impact toughness and average strain rate of concrete.

The relationship between the impact toughness and average strain rate of concrete is as shown in Fig. 17. Formula (6) for the linear relationship was obtained by fitting the impact toughness and average strain rate. According to Fig. 15 and Formula (6), for the four kinds of concrete, when the average strain rate was 50–130 s^−1^, the impact toughness presented a certain strain rate effect and increased with f the average strain rate on the whole. Under the same level of average strain rate, the impact toughness of NC was the biggest, indicating that nano-CaCO_3_ can improve the impact toughness of concrete. As compared with PC, at a low strain rate level, the effect of nano-CaCO_3_ for improving the impact toughness of concrete was not good, while at a high strain rate level, the effect was enhanced obviously, and further advanced with the increase of the average strain rate. Under the same average strain rate level, NSC was between NS and PC for the impact toughness, indicating that nano-CaCO_3_/nano-SiO_2_ co-doping improved the impact toughness of concrete. With the increase of the average strain rate, the gap between NSC and PC continued to widen in impact toughness, and the effect of nano-CaCO_3_/nano-SiO_2_ co-doping for improvement of concrete impact toughness was more and more obvious. Under the same average strain rate, the impact toughness of NS was smaller than that of PC, but with the increase of the average strain rate, the difference between NS and PC in impact toughness became smaller and smaller. The impact toughness of concrete will be reduced by the addition of nano-SiO_2_, but at a high strain rate level, the nano-SiO_2_ had little effect on the impact toughness of concrete.

6 $$ \left\{ {\begin{array}{*{20}c} {R_{{\text{P - PC}}} = 0.00109\overline{\dot{\varepsilon }} + 0.149} \\ {R_{{\text{p - NS}}} = 0.00168\overline{\dot{\varepsilon }} + 0.068} \\ {R_{{\text{p - NC}}} = 0.00505\overline{\dot{\varepsilon }} - 0.077} \\ {R_{{\text{p - NSC}}} = 0.00354\overline{\dot{\varepsilon }} - 0.007} \\ \end{array} } \right. $$

#### Energy dissipation

Energy dissipation $W_{l}$ refers to the ability of concrete to absorb energy under dynamic load, which depends on the strength and deformation amount of material as an integration of the strength and ductility of concrete. In SHPB test of a concrete specimen, under the action of dynamic load, the kinetic energy of the incident bar was eventually converted into the kinetic energy of the transmission bar, the reflected energy of the incident bar and the energy dissipated by the failure of the specimen^32,33^. When the incident energy, transmitted energy, and reflected energy are expressed as $W_{i}$, $W_{t}$, and $W_{r}$ respectively, Formula (7) can be obtained.

7 $$ \left\{ {\begin{array}{*{20}c} {W_{i} = \frac{{A_{e} C_{e} }}{{E_{e} }}\mathop \smallint \limits_{0}^{t} \sigma_{I}^{2} \left( t \right)dt = A_{e} C_{e} E_{e} \mathop \smallint \limits_{0}^{t} \varepsilon_{I}^{2} \left( t \right)dt} \\ {W_{t} = \frac{{A_{e} C_{e} }}{{E_{e} }}\mathop \smallint \limits_{0}^{t} \sigma_{T}^{2} \left( t \right)dt = A_{e} C_{e} E_{e} \mathop \smallint \limits_{0}^{t} \varepsilon_{T}^{2} \left( t \right)dt} \\ {W_{r} = \frac{{A_{e} C_{e} }}{{E_{e} }}\mathop \smallint \limits_{0}^{t} \sigma_{R}^{2} \left( t \right)dt = A_{e} C_{e} E_{e} \mathop \smallint \limits_{0}^{t} \varepsilon_{R}^{2} \left( t \right)dt} \\ \end{array} } \right. $$

where $A_{e}$, $C_{e}$, and $E_{e}$ represent the cross-sectional area, wave velocity and elastic modulus of the bar respectively, and ; $\sigma_{I}$, $\sigma_{T}$, $\sigma_{R} \;{\text{and}}\;\varepsilon_{I}$, $\varepsilon_{T}$, $\varepsilon_{R}$ represent the incident, transmitted, and reflected stresses and strains respectively. Thus, the total energy dissipated by the concrete specimen under dynamic load can be obtained.

8 $$ W_{l} = W_{i} - W_{t} - W_{r} = A_{e} C_{e} E_{e} \mathop \smallint \limits_{0}^{t} \left[ {\varepsilon_{I}^{2} \left( t \right) - \varepsilon_{T}^{2} \left( t \right) - \varepsilon_{R}^{2} \left( t \right)} \right]dt $$

**Figure 18:**
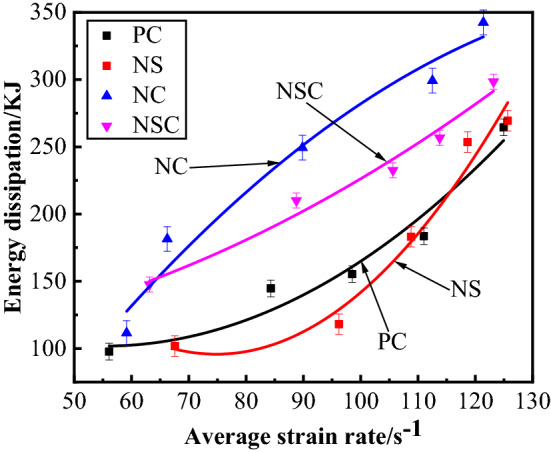
Relationship between energy dissipation and average strain rate of concrete.

The relationship between concrete energy dissipation and average strain rate is as shown in Fig. 18. Formula (9) was obtained through a quadratic fitting of energy dissipation and average strain rate. According to Fig. 16 and Formula (9), for the four kinds of concrete, when the average strain rate was 50–130 s^−1^, the energy dissipation increased with the average strain rate on the whole, showing a strain rate effect. Generally, under the same average strain rate level, the energy dissipation of NC was the biggest, and when the average strain rate was 123.02 s^−1^, the energy dissipation of NC was up to 342.57 kJ. In energy dissipation, NSC was between NC and PC. At a low or medium strain rate (50–115 s^−1^), as compared with PC, the energy dissipation of NS was reduced, and at a high strain rate level (115–130 s^−1^), the energy dissipation of NS was greater than that of PC. Therefore, the nano-CaCO_3_ can improve the energy dissipation of concrete and enhance the capacity of concrete for absorbing energy. At a low or medium strain rate level, the nano-SiO_2_ can weaken the capacity of concrete for absorbing energy, and reduce its energy dissipation as well. At a high strain rate level, the nano-CaCO_3_ had the opposite effect. Although nano-CaCO_3_/nano-SiO_2_ co-doping can also increase the energy dissipation of concrete, the increase rate is smaller than that of nano-CaCO_3_, for the effect of nano-CaCO_3_ for improving the energy dissipation of concrete was partially offset by the nano-SiO_2_.

9 $$ \begin{aligned} W_{{\text{l - PC}}} & = 0.0314\overline{\dot{\varepsilon }}^{2} - 3.471\overline{\dot{\varepsilon }} + 197.59 \\ W_{{\text{l - NS}}} & = 0.0723\overline{\dot{\varepsilon }}^{2} - 10.807\overline{\dot{\varepsilon }} + 499.83 \\ W_{{\text{l - NC}}} & = - 0.0233\overline{\dot{\varepsilon }}^{2} + 7.491\overline{\dot{\varepsilon }} - 233.78 \\ W_{{\text{l - NSC}}} & = 0.0125\overline{\dot{\varepsilon }}^{2} + 0.0325\overline{\dot{\varepsilon }} + 98.49 \\ \end{aligned} $$

#### Impact failure mode

**Figure 19:**
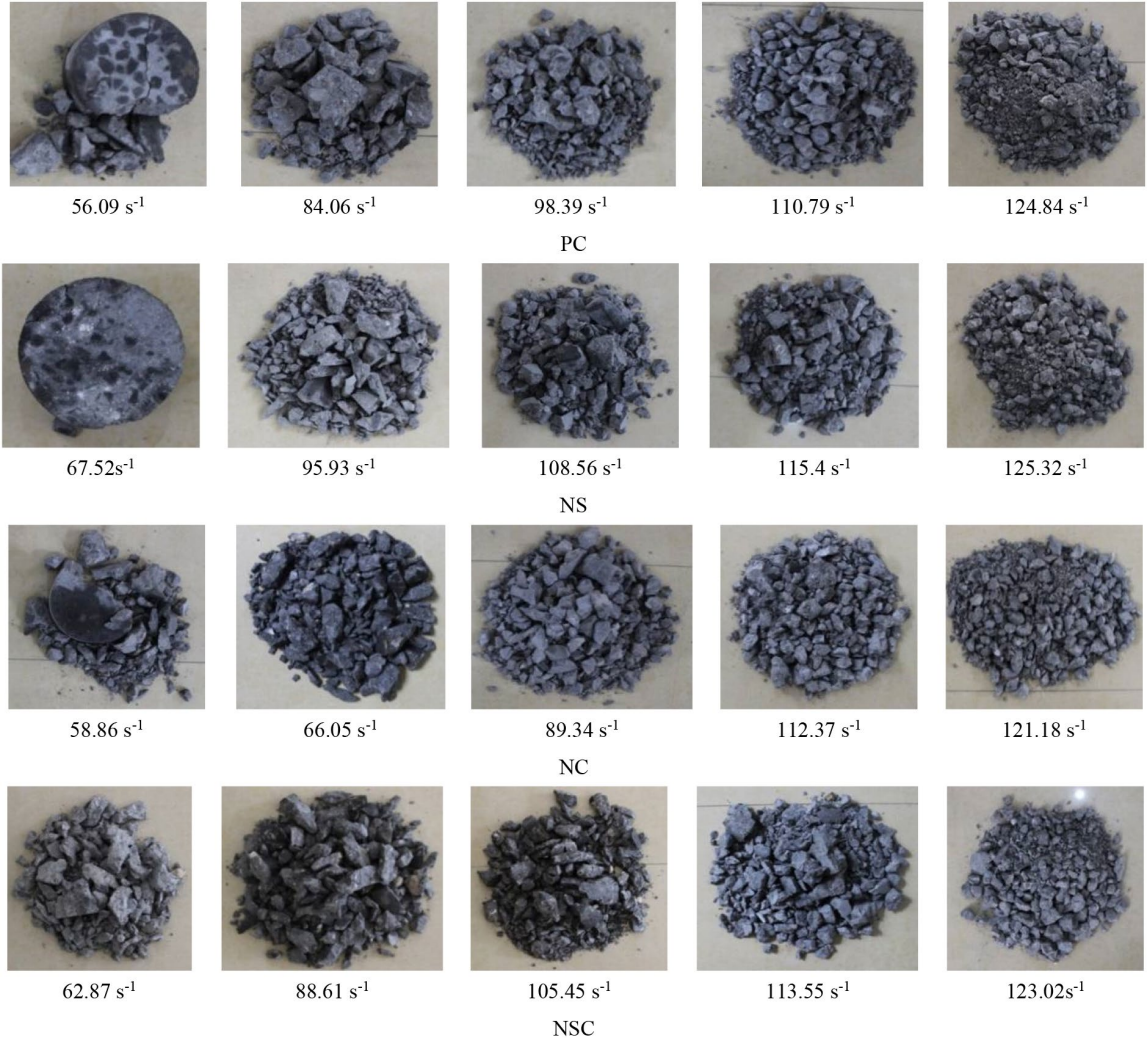
Failure mode of concrete.

In the SHPB test, the concrete specimens were damaged under a certain dynamic load, and the failure modes for each group of concrete specimens were as shown in Fig. 19. Obviously, for the four kinds of concrete, with the increase of the average strain rate, the damage was more obvious. Although the failure modes of concrete specimens were various at different average strain rate levels, they can be classified as three types. (1) Edge damage. This type of failure modes refers to the fact that the specimen remains intact mostly, and there is only a little damage at the edge of the specimen, which generally occurs at a low strain rate level. For example, for a failure mode when the average strain rate of NC was 67.52 s^−1^, even though the specimen remained intact without obvious fracture trace after the dynamic loading, a crack had been formed inside the specimen, and the specimen had been destroyed essentially. (2) Fragmentation. This type of failure modes is more serious than the edge failure, and the specimen does not break into several pieces after bearing dynamic load, e.g., the damage of PC at an average strain rate of 84.06 s^−1^. (3) Crushing destruction. There is almost no large fragment for this type of failure modes, and the specimen is generally broken into fragments. In general, the failure modes at a high strain rate are all of this type, e.g., the damage of NSC at an average strain rate of 123.02 s^−1^.

## Analysis on the microscopic mechanism

### Micromorphology

**Figure 20:**
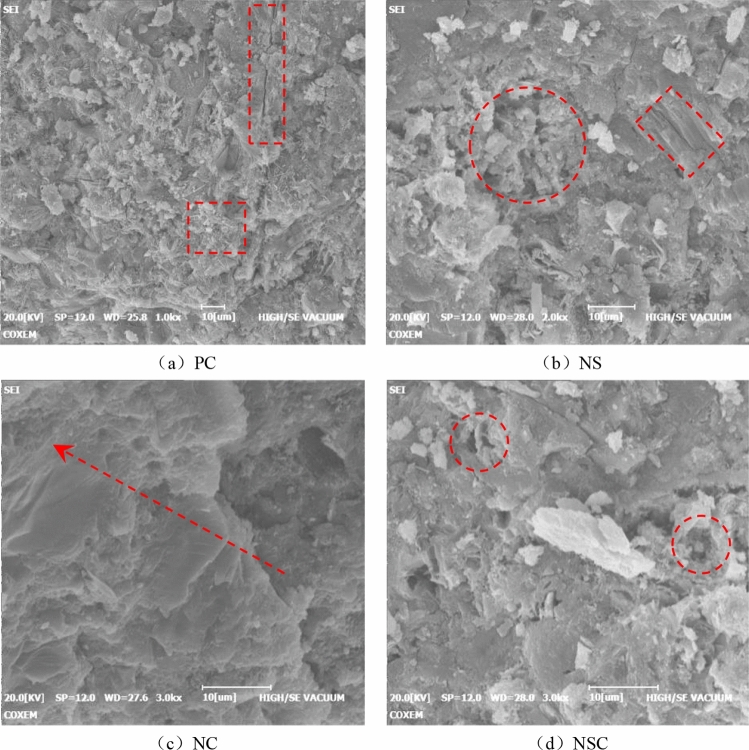
Micro-morphology of concrete.

The addition of nanoparticles can improve the structural composition of concrete at a microscopic level, thus affecting the static and dynamic mechanical properties of concrete. The micromorphology of concrete is as shown in Fig. 18. According to Fig. 20a, the internal structure of PC was not uniform, but there were a large number of crystal clusters, acicular crystals and obvious cracks, and the structure was relatively loose. As shown in Fig. 20b, there was an obvious weak area in NS, where voids were formed, and the voids were surrounded by large crystals with clear outlines. As shown in Fig. 20c, there were obvious differences between NC, PC and NS in microstructure. On the whole, the internal structure of NC was very uniform without significant weak area, showing a cascading structure, and there were no clusters or needle-like harmful crystals on the crystal layer. According to Fig. 20d, the microscopic morphology of NSC was similar to that of NC, and there were weak areas, but for the weak areas, the range was significantly reduced, and there was no large crystal with clear outline around the weak areas.

The nano-SiO_2_ is of an amorphous vitreous structure with a large specific surface area and a large number of unsaturated bonds on the surface, for which large amounts of water may be adsorbed on the surface of the particles, showing a high chemical activity. In the early stage of cement hydration reaction, the hydration product Ca(OH)_2_ may react with nano-SiO_2_ to form a high strength C–S–H gel with flocculent structure, thus reducing the production of Ca(OH)_2_ and refining the crystal structure of Ca(OH)_2_^34^. However, after a certain degree of hydration, the free water in contact with the cement particles in the concrete will decrease rapidly. Without enough water to react with the cement particles, the hydration reaction cannot be complete, thus forming an obvious weak area in the concrete, which leads to the degradation of static and dynamic mechanical properties of NS. The nano-CaCO_3_ particles in concrete may adsorb free CA^2+^ easily for its high surface energy, thus accelerating nucleation of Ca(OH)_2_ crystals around it, improving the enrichment and arrangement of Ca(OH)_2_ crystals, and promoting the crystals to fill the weak areas of concrete^35^. Furthermore, with nanometer CaCO_3_ as the core, the C–S–H gel is easy to form a new columnar network structure around the particles, which intersects with the original microstructure to fill the harmful voids, thus increasing the compactness of concrete, and strengthening the static and dynamic mechanical properties of NC. In nano-SiO_2_/nano-CaCO_3_ co-doping, the nano-SiO_2_ and the nano-CaCO_3_ may work together in concrete_._ Specifically, the nano-SiO_2_ leads to the formation of obvious weak areas in concrete, while the nano-CaCO_3_ promotes the filling of weak areas by Ca(OH)_2_ crystals. Under the combined action of the two, there may be still some weak areas in NSC, and so, NSC is between NS and PC for the mechanical properties.

### Pore size Distribution

**Figure 21:**
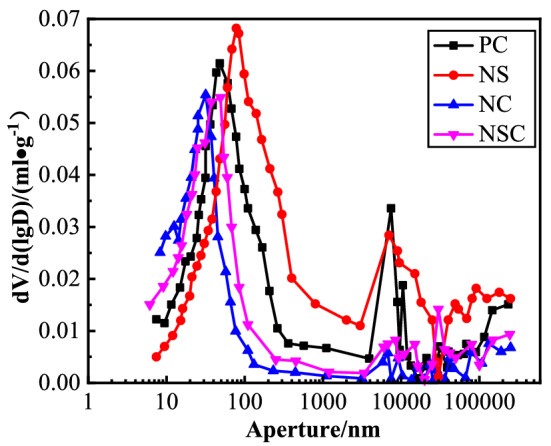
Pore size distribution curve of concrete.

The pore size distribution curve of concrete is as shown in Fig. 21. Obviously, the pore size of NC was unimodal distribution, and of PC, NS and NSC was roughly bimodal distribution. Peak 1 of PC pore size distribution curve appeared near 100 nm, and Peak 2 near 10,000 nm. The overall pore size distribution of NC was lower than of PC, and the pore size of NC tended to be small, mainly in the range of 10–100 nm, with relatively few large pores. The addition of nano-CaCO_3_ can optimize the pore size distribution in concrete, refine the pore structure, and reduce large pores. The pore size distribution of NS was bimodal, similar to that of PC. Peak 1 of the pore size distribution curve shifted to large pore size, while for Peak 2, the range was expanded. The addition of nano-SiO_2_ degraded the pore structure of concrete, decreased the pore size, and increased the large pores^36^. NSC was between PC and NS in pore size distribution. Peak 1 on the pore size distribution curve was between Peak 1 of PC and Peak 1 of NS. As compared with PC, Peak 2 of NSC shifted towards large pore size, but the range was obviously reduced. The addition of nano-CaCO_3_ can optimize the pore structure of concrete, and so, the static and dynamic mechanical properties of NC were improved, while nano-SiO_2_ may degraded the pore structure of concrete, and thus, the static and dynamic mechanical properties of NS was compromised.

## Conclusion

With HYY series hydraulic servo test system and Φ100 mm split Hopkinson pressure bar (SHPB) test system, the static and dynamic mechanical tests under different strain rates were carried out, and the effects of nano-SiO_2_ and nano-CaCO_3_ on the static and dynamic mechanical properties of concrete were explored as well. According to relevant analysis, single-doped nano-CaCO_3_ and co-doped nano-CaCO_3_/nano-SiO_2_ can improve the static and dynamic mechanical properties of concrete, while single-doped nano-SiO_2_ will weaken the static and dynamic mechanical properties of concrete. Overall, the findings are as follows:

1. Single-doped nano-CaCO_3_ and co-doped nano-SiO_2_/nano-CaCO_3_ can improve the compressive strength, flexural strength, splitting tensile strength and elastic modulus of concrete. Single-doped nano-SiO_2_ may weaken the compressive strength, flexural strength and splitting tensile strength of concrete, but can increase the elastic modulus of concrete.
2. The dynamic compressive strength, impact toughness, energy dissipation and impact failure modes of concrete have an obvious strain rate effects. With the increase of strain rate, the dynamic compressive strength, impact toughness and energy dissipation of concrete increase gradually, and the damage degree becomes more significant.
3. Under the same strain rate level, single-doped nano-CaCO_3_ can improve the dynamic compressive strength, peak strain, impact toughness and energy dissipation of concrete, but may reduce the dynamic elastic modulus of concrete, while single-doped nano-SiO_2_ may decrease the compressive strength, peak strain, impact toughness and energy dissipation of concrete, but can increase the dynamic elastic modulus of concrete.
4. For the static and dynamic mechanical properties, NSC is between PC and NC. On the whole, the co-doped nano-SiO_2_/nano-CaCO_3_ can improve the static and dynamic mechanical properties of concrete, but the effect is weakened as compare with single -doped nano-CaCO_3_.
5. Single-doped nano-CaCO_3_ can advance the compactness of concrete, improve the weak area of concrete, and optimize the pore size distribution of concrete; single-doped nano-SiO_2_ may cause obvious weak areas inside concrete, and degrade the pore structure deterioration; co-doped nano-SiO_2_/nano-CaCO_3_ can make the two nanomaterials work together in concrete.
